# Characterizing the Volatile and Sensory Profiles, and Sugar Content of Beeswax, Beebread, Bee Pollen, and Honey

**DOI:** 10.3390/molecules26113410

**Published:** 2021-06-04

**Authors:** Małgorzata Starowicz, Paweł Hanus, Grzegorz Lamparski, Tomasz Sawicki

**Affiliations:** 1 Department of Chemistry and Biodynamics of Food, Institute of Animal Reproduction and Food Research of the Polish Academy of Sciences, 10 Tuwima Street, 10-748 Olsztyn, Poland; m.starowicz@pan.olsztyn.pl; 2 Department of Technology and Plant Product Quality Assessment, University of Rzeszów, 4 Zelwerowicza Street, 35-601 Rzeszów, Poland; phanus@ur.edu.pl; 3 Sensory Laboratory, Institute of Animal Reproduction and Food Research of the Polish Academy of Sciences, 10 Tuwima Street, 10-748 Olsztyn, Poland; g.lamparski@pan.olsztyn.pl; 4 Department of Human Nutrition, Faculty of Food Sciences, University of Warmia and Mazury in Olsztyn, 45f Słoneczna Street, 10-718 Olsztyn, Poland

**Keywords:** aroma composition, bee products, PLS, sugar content, QDA profile

## Abstract

Bee products are a well-known remedy against numerous diseases. However, from the consumers’ perspective, it is essential to define factors that can affect their sensory acceptance. This investigation aimed to evaluate the volatile and sensory profiles, and sugar composition of beeswax, beebread, pollen, and honey. According to the HS-SPME/GC-MS results, 20 volatiles were identified in beeswax and honey, then 32 in beebread, and 33 in pollen. Alkanes were found to dominate in beeswax, beebread, and pollen, while aldehydes and monoterpenes in honey. In the case of sugars, a higher content of fructose was determined in beebread, bee pollen, and honey, whereas the highest content of glucose was assayed in beeswax. In the QDA, the highest aroma intensity characterized as honey-like and sweet was found in honey, while the acid aroma was typical of beebread. Other odor descriptors, including waxy, pungent, and plant-based aromas were noted only in beeswax, honey, and pollen, respectively.

## 1. Introduction

Honeybee products, including beeswax, beebread, bee pollen, and honey, have been intensively studied for their efficacy against some diseases [1,2,3,4]. However, aroma and flavor determination is nowadays also an important aspect considering the consumers’ acceptance of varied food products, and thus the potential industrial application of raw materials in food design. Kaškoniene and Venskutonis [5] collected examples of research proving that the volatile composition of honey is strictly related to its botanical origin.

Honey is the best-known bee product. It is broadly used in every household and its consumption has been declared by over 95% of the Polish consumers [6]. Consumers typically prefer light-colored honey and often choose multiflorous, linden, acacia, rapeseed, and honeydew honeys [6]. Honey has a very pleasant aroma and sweet taste [7], therefore it is used to sweeten meals (as declared by about 70% of consumers). An increasing number of consumers appreciate the therapeutic effects of other bee products with the wide application in apitherapy, but also their sensorial properties. Bee pollen contains about 250 substances, two-thirds of which are carbohydrates, and ~94% of these carbohydrates are monosaccharides (mainly fructose, glucose, and sucrose) [8,9]. Moreover, beebread is a unique product, while beeswax is synthetized from honey sugars and represents a complex mixture of esters, hydrocarbons, free fatty acids, alcohols, and other substances [10]. Beeswax is used in the European Union as a glazing agent, food additive labeled as E901, and also widely as a carrier for flavors and coatings [11,12]. The bee products, especially honey, are often used as meal sweeteners, due to a high content of carbohydrates. The sugar composition of honey depends mainly on its botanical and geographical origins, and is affected by climate, processing, and storage [13]. Moreover, the concentration of fructose and glucose, as well as the ratio between them are useful indicators in the classification of honey types [14].

Honey and other bee products are used in food technology due to their valuable chemical composition and sensory characteristics. According to Tomczyk et al. [15], spray-dried honey can be an additive to isotonic drinks, improving their taste and colour. In addition to its wide use in the cosmetics industry, beeswax is also used in food technology to protect ripened cheeses as a polishing agent or edible coating mixed with polylactic-acid (PLA) [16]. Moreover, the addition of bee pollen improves the nutritional, functional, and sensory properties of food [17]. The use of bee pollen in white wine production increases the number of volatile compounds in wine. The addition of up to 1 g/L of multifloral pollen significantly affects the perception of fruit and floral notes in wines [18].

The collected literature allows us to conclude that beeswax and beebread are the least characterized of bee products. Therefore, the aim of this research was to study the volatile and sensorial profiles, and sugar content of beeswax, beebread, bee pollen, and honey of the same origin and from the same batch.

## 2. Results and Discussion

### 2.1. The Profile of Volatiles Compounds in Bee Products

There is only scarce information about the profiles of volatile compounds of bee products. Most studies dedicated to the volatile profiles refer to honey [19,20]. While, the research on the characteristics of volatiles in beeswax, bee pollen, and beebread mainly concern their use as additives, which allows enriching the flavor and aroma profile, and bioactive properties of various final products [21,22]. Thus, this is the first research presenting the profile of volatile compounds of four different bee products (honey, beebread, bee pollen, and beeswax).

A total of 55 volatile compounds identified in this study are presented in Table 1. They include 15 alkanes (volatile nos. X1, X2, X3, X4, X5, X6, X7, X8, X9, X11, X16, X17, X18, 25, and 27; refer to Table 1), eight compounds from monoterpenes (nos. 10, 20, 22, 30, 39, 41, X47, and X50), seven alcohols (nos. X23, X29, X33, X38, X43, X51, and X52), also seven acids (nos. X31, X45, X46, X48, X49, X53, and X55), five aldehydes (nos. X13, X21, X28, X34, and X37), two esters (X26 and X54), two ketones (24 and 36), and two compounds from benzenes (nos. X15 and X19), as well as disulfides (compound no. X12), sulfoxides (X14), furans (X32), oxygenated hydrocarbon (X36), pyrroles (X40), and lactones (X44), represented by one molecule. 

Nonanal (X28), furfural (X32), and benzaldehyde (X37) were detected in all samples of been products (Table 1). In addition, 2,4-Dimethyl-heptane (X1), 4-methyl-octane (X2), 2-methyl-nonane (X5), 4-methyl-decane (X7), dodecane (X17), octanal (X21), and tetradecane (X27) were found in beeswax, beebread, and bee pollen, while, acetic acid (X31) and hexanoic acid (X49) were present only in beebread, bee pollen, and honey.

As shown in Table 1, the total sum of volatiles was detected within the range of 138.29–267.13 ppm (presented as peak areas). The highest relative content of these compounds was detected in bee pollen (267.13 ± 8.32). However, no statistical differences were found between bee pollen and beebread (*p* < 0.05). In contrast, the lowest sum of volatiles was determined in beeswax (138.29 ± 5.73). The sum of volatiles could be ranked as follows: Bee pollen ≥ beebread > honey > beeswax. However, the order of the number of volatiles in bee products was: Bee pollen (33 compounds) > beebread (32) > honey = beeswax (20). In addition, 2,4-dimethyl-heptane (X1) was the dominant compound in beeswax (18.9% of total volatile amount) and bee pollen (13.7%). Furthermore, 4-methyl-octane (X2; 13.8%; *p* < 0.05) also dominated in bee pollen, while benzaldehyde (X37) had a significantly higher percent contribution in the volatile profile in honey (26% of total volatiles). In case of the beebread, four compounds were prevailing, including three from the group of alkenes (X6, X9, and X25) and one from the group of furans (X32). These major compounds accounted for approximately 38% of the total peak area in the beebread. As mentioned above, several volatiles were found only in one bee product (Table 1). Nine volatiles (X20, X22, X30, X39, X41, X42, X48, X50, and X53) were only present in honey, six (X14, X23, X26, X34, X47, and X54) in bee pollen, while, five (X10, X29, X35, X38, and X43) volatiles were only present in beeswax. 

The richest profile of volatiles was detected in the bee pollen (33 volatiles), therefore they are listed in Table 1. In contrast, a study conducted by Keskin and Özkök [23], showed only 25 compounds in the bee pollen from Turkey. Tetradecane (X27) was the common compound identified in the Turkish bee pollen and bee products analyzed in our study. A small number of volatiles (propanedioic acid, benzoic acid, phenylacetic acid, 1-dodecene, hexadecenoic acid, 9,12-octadecadienoic acid, 9,12,15-octadecatrienoic acid, and nonadecanoic acid) were also detected in the bee pollen from Brazil [24]. On the other hand, the largest number of volatiles (137 compounds) was detected in the bee pollen from the mid-north region of Brazil [25]. Identification of such a large number of volatile compounds was feasible due to the use of many extraction methods (micro-hydrodistillation, dynamic headspace, ultrasound-assisted extraction, and solid phase microextraction). Only the headspace solid phase microextraction method alone (HS-SPME, a method that was also used in our study) allowed determining a high number of volatiles (84) in the bee pollen samples [25]. On the other hand, the compound number detected may be related to the region from which the samples were collected. The previous studies have also shown a relationship between the profile of volatile compounds in bee products and their region of origin [19]. Kaškonienė et al. [26] also found a high number of volatiles (103) in bee pollen collected in Lithuania. However, only 26 of them were in the amounts exceeding 1% of total volatiles in the sample. The following volatiles detected by these authors in bee pollen: Hexanal (X13), styrene (X19), benzaldehyde (X37), 6-methyl-5-hepten-2-one (X24), octanal (X21), (*E*,*E*)-3,5-octadien-2-one (X36), nonanal (X28), dodecane (X17), and 1-tridecene (X25) were also identified in our research. One of the main compounds in the bee pollen from Lithuania was styrene, with the content ranging from 19.6 to 27% of total volatiles [26]. In our study, its relative content was only 1% of the total volatiles of bee pollen, which may indicate that bee pollen from Poland may be less contaminated. According to the available literature, styrene in bee pollen can come from plastic bags [27], but it may also exist naturally or could be produced during enzymatic synthesis since bee pollen is produced by bees with the use of their saliva [28]. 

**Figure 1:**
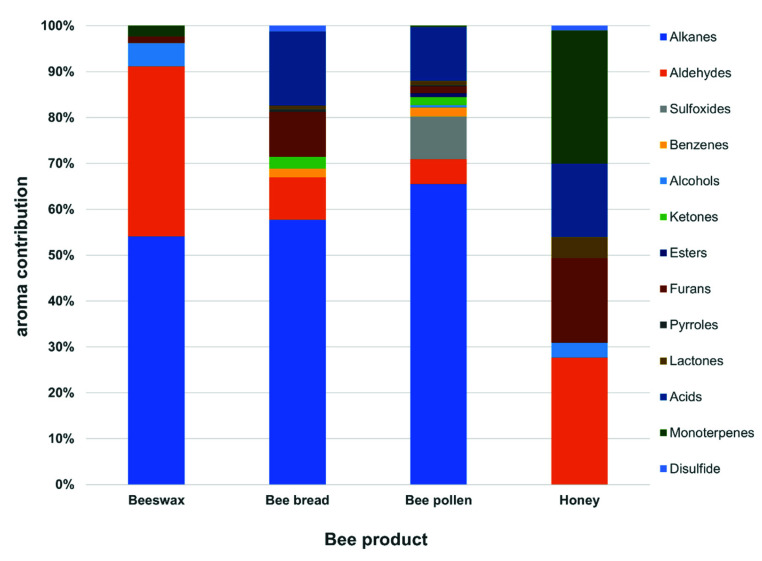
Chemical classes contribution of volatile compounds detected in bee products.

The bee pollen possessed the richest profile of chemical classes among other bee products (12 from 13 chemical groups). Its volatile profile included 13 alkanes, five aldehydes, two acids, two benzene derivatives, two ketones, two esters, and one compound from other groups (sulfoxides, alcohols, pyrroles, furans, lactones, and monoterpenes). The main chemical group in bee pollen was represented by alkanes (65.5% of total volatiles; Figure 1). Furthermore, the second most dominant chemical group included acids (11.8%), followed by sulfoxides (9.3%), and aldehydes (5.4%), whereas the percent contribution of other groups was below 2%. In comparison, the bee pollen from Brazil contained only two groups of compounds, i.e., esters (seven compounds) and alkane [24]. The other Brazilian bee pollen was characterized by compounds from six chemical groups, while thirteen groups were identified in our study. Brazilian bee pollen was characterized by higher numbers of esters, ketones, and hydrocarbons, while lower numbers of aldehydes and the same number of alcohols in comparison to our study. Results of a study by Lima Neto et al. [25] did not show volatiles from the groups of alkanes, monoterpenes, disulfide, sulfoxide, benzenes, acids, furans, lactone, and pyrroles in pollen samples. On the other hand, terpenoids which were detected in the Brazilian bee pollen were not found in bee pollen from our study. A different number of volatiles detected may result from the botanical origin [20], time of harvest, and method of extraction [25]. 

The next bee product with the high number of volatile compounds (32) was beebread. It contained 15 alkanes (X1, X2, X3, X4, X5, X6, X7, X8, X9, X11, X16, X17, X18, X25, and X27), four aldehydes (X13, X21, X28, and X37), five acids (X31, X45, X46, X49, and X55), two benzenes (X15 and X19), two ketones (X15 and X19), and others (disulfides, furans, pyrroles, and lactones). The results indicated a high contribution of alkanes in beebread, representing 47% of all identified compounds, and their 57.7% contribution to the aroma profile (Figure 1). Volatiles detected in the beebread belonged to nine out of the 13 chemical groups classified in the bee products. Moreover, it was the only sample from the tested bee products whose volatile profile was common with the profiles of the other samples, probably since it is a combination of honey and pollen [3,29]. Furthermore, our study showed that the volatiles present in bee pollen played a more important role in creating the profile of volatile compounds of beebread than these of honey.

To the best of our knowledge, there is paucity of data on the profile of volatile compounds in beebread. Results of a study by Kaškonienė et al. [30] also showed 32 volatiles in beebread from Lithuania. Comparing the beebread from Poland and Lithuania, the same 11 volatiles (2,4-dimethyl-heptane, nonane, decane, dimethyl disulfide, dodecane, octanal, nonanal, acetic acid, furfural, benzaldehyde, and hexanoic acid) were present in both samples. As mentioned above, the main compounds in beebread in our research were decane (X6), furfural (X32), 2,6,11-trimethyl-dodecane (X9), and 1-tridecene (X25), each contributing approximately 9% to the total volatiles (Table 1). However, the beebread from Lithuania had high contents of dimethyl sulphide, 1-heptadecene, acetic acid, nonane, and furfural, accounting for 20.0%, 13.9%, 13.4%, and 9.8% of the total volatiles, respectively [30]. Furfural was detected in the samples from Lithuania and Poland. Moreover, its content in both beebreads was the same (9.8% of the aroma profile). It is common knowledge that the furfural concentration is associated with the heat treatment [31], therefore, the high contribution of this compound may depend on the extraction method (SPME). The next compound with a high contribution in the beebread from Lithuania was acetic acid. However, its content in the sample from Poland was lower by 6.3%. The source of acetic acid in beebread is unknown, but there are two possibilities. As mentioned above, the beebread is a mixture of honey and bee pollen, which undergoes fermentation by enzymes of the bee’s saliva and Lactobacillus [32], resulting in the formation of acetic acid. On the other hand, acetic acid—as a biological metabolic intermediate—occurs naturally in plant juices [33], which may also be collected by bees. 

In honey, we identified 20 compounds in total, including: six acids (X31, X45, X48, X49, X53, and X55), six monoterpenes (X20, X22, X30, X39, X41, and X50), three volatiles from the group of alcohols (X33, X51, and X52), two aldehydes (X28 and X37), and one compound from each of the following groups: Disulfides (X12), furans (X32), and lactones (dihydro-4-methyl-2(3*H*)-furanone). These results concerning the detected chemical families are consistent with findings reported by other authors [20]. On the other hand, a recent study demonstrated a higher number of volatiles (42 compounds) in honey from China, which included 14 aldehydes, 12 ketones, seven alcohols, three acids, three pyrazines, one ether, one ester, and one terpene [34]. In Polish honey, we did not detect volatiles from ketone, pyrazine, ether, and terpene groups, but were identified compounds from groups of: Monoterpenes, disulfides, and furans, which were not detected in the Chinese samples. Moreover, the other research also showed more volatiles (60) detected in unifloral Chinese kinds of honey [35].

The contribution of the sum of aldehydes in the honey accounted for over 27%. However, the contribution of monoterpens was higher than that of aldehydes and was estimated at 29% (Figure 1). In the case of acids, the major compound was 2/3-methyl-butanoic acid accounting for 51.5% and 7.4% of the total acids and volatiles, respectively. The major monoterpene turned out to be linalool, which accounted for 49.4% and 14.3% of total monoterpenes and aroma profile, respectively. However, the results presented in Table 1 indicate that the major volatile in the honey was benzaldehyde. Its contribution in the tested sample was approximately 26% of the total volatiles. Moreover, it was present in all bee products, with the highest relative content detected in honey, being higher by 88.5%, 89.4%, and 91.8% than in bee pollen, beebread, and beeswax, respectively (Table 1). Furfural was also present in the honey in a relatively high amount (18.4% of peak areas). It was also detected in the bee pollen, beebread, and beeswax, while as in the case of benzaldehyde, the highest contribution of this compound was determined in honey. 

The composition of volatiles of beeswax was significantly different than the profile of volatile compounds in honey, bee pollen, and beebread (Table 1). The HS-SPME-GC/MS analysis showed the presence of only five of the thirteen classes of chemical compounds detected in bee products. As mentioned above, a total of 20 compounds were identified in beeswax, including eight alkanes (X1, X2, X3, X5, X7, X17, X18, and X27), six alcohols (X29, X33, X38, X43, X51, and X52), four aldehydes (X21, X28, X35, and X37), and another two volatiles containing one monoterpene (X10) and one furan (X32). In the beeswax, the alkanes were the dominant group of compounds with 54.1% of contribution to the aroma profile (Figure 1). The next group of volatiles was aldehydes, which accounted for 37.1% of the aroma profile. Moreover, alcohols with the higher number of compounds (6) than aldehydes (4) were characterized by a lower contribution (5.0%) to the sum of volatiles detected in beeswax. The beeswax had the highest content of 2,4-dimethyl-heptane (18.9% of total peak area). Moreover, octanal and nonanal were the major contributors to the volatile composition in beeswax, accounting for 13.4% and 11.0%, respectively. On the other hand, the content of 4-methyl-decane (X7), α-pinene (X10), nonadecane (X18), tetradecane (X27), 1-heptanol (X29), furfural (X32), 2-ethyl-1-hexanol (X33), benzaldehyde (X37), 1-octanol (X38), 1-nonanol (X43), benzyl alcohol (X51), and phenylethyl alcohol (X52) were below 3.2%. Although beeswax is a precious product and its aroma is one of its incredibly privileged properties, unfortunately, only one team has undertaken an attempt of analyzing the profile of its volatile compounds so far. The study by Ferber and Nursten [36] showed the presence of 48 volatiles, divided into four different chemical classes. The larger number of volatile compounds identified in the above cited study compared to our research was due to the fact that the cited authors used two columns of different polarities. Moreover, the composition of beeswax is influenced by the genetic determinants of the bee colony and environmental factors [37].

### 2.2. Sugar Contents in Bee Products

The results of the sugar content analysis in bee bread, bee pollen, beeswax, and honey are provided in Table 2. Fructose, glucose, and sucrose contents were determined, and the total sugar content and fructose/glucose (F/G) ratio were calculated, as well. Glucose and fructose were the major components in the investigated bee products. The highest total sugar content was determined in honey (54.02 g/100 g), then pollen (25.63 g/100 g), and bee bread (16.81 g/100 g), and the lowest one in beeswax (2.09 g/100 g). Glucose and fructose contents in honey were lower than these noted by Szczęsna et al. [38] for rape honey, i.e., 37.6 and 37.3 g/100 g, respectively. Fructose, with the mean content of 35.91 g/100 g, followed by glucose, with the mean content of 35.00 g/100 g, were also detected in floral honey samples in the study by Kirs et al. [39]. The differences in sugar content could be related to the various floral/geographical origin of honey from the cited and our study. However, the range of glucose and fructose contents determined in our study were similar to those presented for multifloral light honey [40]. Moreover, the analyzed honey met the requirements set out in the European regulations concerning the sum of fructose and glucose. Furthermore, the honey sample did not exceed the permissible levels for sucrose [11,41]. Only a low content of sucrose was detected at 0.10 g/100 g for honey, at 0.11 for beebread, and at 0.01 for bee pollen. No sucrose was reported in beeswax in our study. This is in agreement with the data reported by Bertoncelj et al. [42], who determined the contents of sucrose in bee pollen only in the range of 0.05–0.28 g/100 g.

The fructose/glucose (F/G) ratio is a standard parameter launched in many research dedicated to bee products. Therefore, the F/G ratio was also examined in our study and ranged from 2.26 (in beebread) to 0.03 (in beeswax) (Table 2). Its highest value was noted in beebread, followed by bee pollen (F/G = 1.22). This data is similar to those reported by Martins et al. [43] and Kalaycıoglu et al. [44], who calculated the F/G ratio in the range of 1.01–2.24 for bee pollen from Brazil, and in the range of 1.12–2.53 for bee pollen from Turkey, respectively. The F/G of honey analyzed in our study reached 1.05 and was similar to that noted by Szczęsna et al. [45] and Kirs et al. [39]. Moreover, Kirs et al. [39] emphasized that the F/G ratio could be a good indicator of honey origin. They reported that the mean F/G = 1.03 could be a marker of floral honey, which is correct in the context of our multiflorous honey. The lowest F/G value was determined for beeswax, since its major sugar is glucose, which is less sweet than fructose. Therefore, the content of sugars is important not only from the viewpoint of product authenticity, but also regarding consumer acceptance, as consumers definitely prefer more sweet products (in both taste and aroma). 

### 2.3. QDA Profile of Bee Products in the Aspect of Color and Odor Quality

The QDA method of sensory profiling has already been used to determine the properties of honey samples [46,47]. The crucial points in this method include an appropriate training of the sensory panel and the choice of descriptors and standards related to them. Therefore, in the primary step, panelists prepare definitions of odor terms and choose reference materials, which help them identify the selected odors. In this case, honey-like was defined as an aromatic impression related to honey, and the standard used in this case was an essence on the tissue paper le nez du café no. 19 honeyed; sweet was described as a delicate sweet note characteristic of cotton candy (cotton candy); acid as an odor associated with organic acid, e.g., organic acids (lemon slice); pungent as an intensive odor of spices (black pepper); waxy as an odor characteristic of beeswax (beeswax); and plant-based as an odor related to plant material.

**Figure 2:**
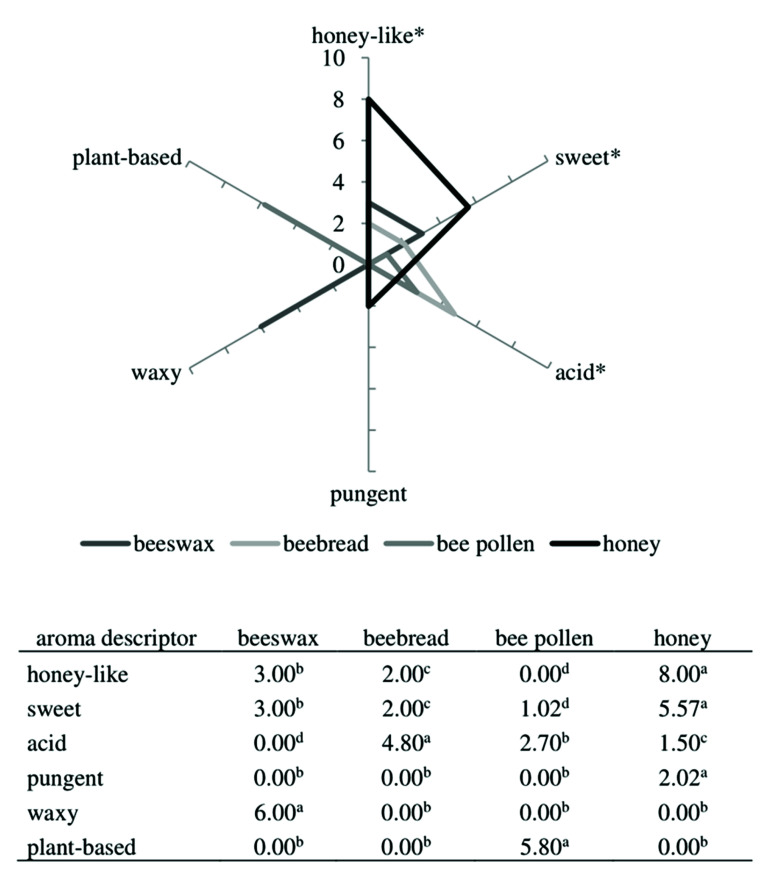
Spider diagram of mean scores of aroma attributes of bee products. Different letters in the same row and “*” in the graph mean significantly different (*p* < 0.001).

The results of QDA are presented in Figure 2. According to the scores given by the sensory panel to the color of bee products, beeswax was the less yellow product with a value of 3.53 arbitrary units (au), in comparison to beebread (9.00 au), honey (7.00 au), and bee pollen (5.00 au). Moreover, the sensory panel identified six odor descriptors for bee products: Honey-like, sweet, acid, pungent, waxy, and plant-based. Honey received the highest score for honey-like odor (2–3 times higher compared to beeswax and beebread), and had the sweetest aroma compared to the other bee products. This result is in agreement with findings from our previous study (data not published), indicating that multiflorous honey possesses the sweetest and honey-like odor compared to other types of honey. Castro-Vázquez et al. [46] defined the main honey descriptors as floral and fruity. However, they can be contributed to sweet and honey-like sensory markers. In the bee pollen, panelists did not note the honey-like aroma, but its aroma was described using attributes of sweet, acid, and plant-based. Moreover, Sipos et al. [48] characterized the aroma of bee pollen as sour/acid at the same level as in our study. Furthermore, in the present study, beebread exhibited the highest intensity for the attributes of acid aroma, whereas in beeswax acidity was not recognized at all. Other odor descriptors, including waxy, pungent, and plant-based were noted only in beeswax, honey, and pollen, respectively. The waxy in beeswax and plant-based in pollen received the high scores of 6.00 and 5.80 au, respectively, which might be deemed sensory markers of these two products. 

In a future perspective, the sensory analysis could be helpful to choose bee products with the highest rate of aroma notes and with high consumer acceptability. Therefore, the sugar content and profile of volatile compounds could be correlated with sensory descriptors of bee products. The relationship between each analysis is described in the next step of this manuscript—the PLS analysis.

### 2.4. The PLS Analysis

**Figure 3:**
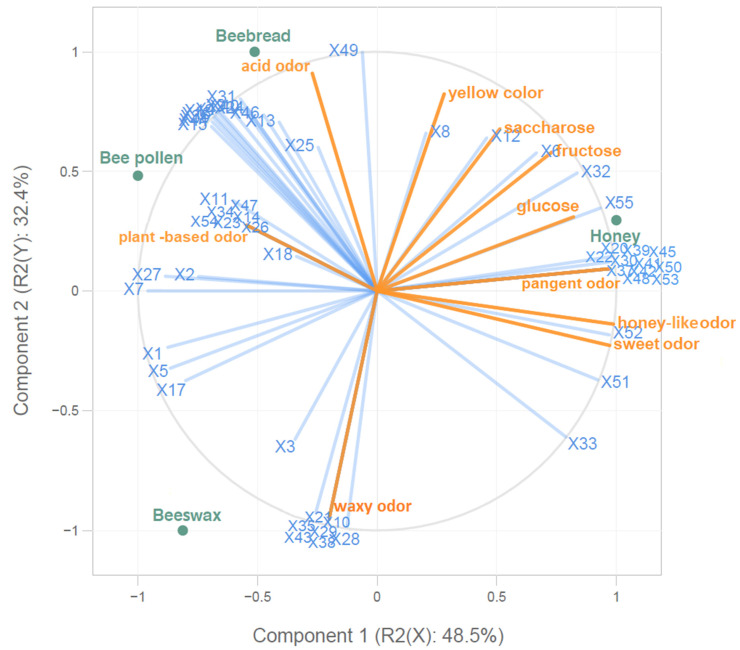
PLS correlation analysis of samples, between aroma compounds and sensory attributes; X1–X55 represent the number of compounds identified in the bee products (Table 1).

The partial least squares (PLS) analysis was performed to evaluate the overall association between the bee products, aroma compounds, and sensory descriptors (Figure 3). The analysis performed explained 80.9% of the total variation. Beebread was located on the top left and was positively associated with one sensory descriptor (acid odor) and eleven volatiles (3,8-dimethyl-decane, 2,6,11-trimethyl-dodecane, hexanal, 1-(3,3-dimethylbutyl) benzene, 2,6,10-trimethyl-tetradecane, 6-methyl-5-hepten-2-one, acetic acid, (*E*,*E*)-3,5-octadien-2-one, 1-ethyl-1H-pyrrole-2-carbaldehyde, butyrolactone, and 2-methyl-hexanoic acid). Most of the compounds that correlated with beebread were characterized by green, fruity, citrus-like, and/or pungent aroma. Moreover, bee pollen was located in the upper left quadrant, and was moderately correlated with one volatile compound (5-methyl-decane). On the other hand, honey was located on the top right. It was positively correlated with twelve volatile compounds (*o*-cymene, *p*-cymene, *cis*-linalool oxide, furfural, benzaldehyde, linalool, hotrienol, dihydro-4-methyl-2(3*H*)-furanone, 2/3-methyl-butanoic acid, *p*-cymene-8-ol, 3-methylvaleric acid, and heptanoic acid), but mostly with nonanoic acid. Moreover, honey was positively correlated with the sensory descriptor of pungent odor, which may be positively influenced by the presence of *o*-cymene, *p*-cymene, *cis*-linalool oxide, benzaldehyde, linalool, hotrienol, dihydro-4-methyl-2(3*H*)-furanone, 2/3-methyl-butanoic acid, *p*-cymene-8-ol, 3-methylvaleric acid, and heptanoic acid, and positively correlated with the concentration of sugars, but mostly with glucose. The highest correlation was achieved between waxy odor and molecules of α-pinene, octanal, nonanal, 1-heptanol, 1-octanol, and 1-nonanol, while a lower correlation was observed in the case of waxy odor and nonane. Honey and beebread were positively correlated with furfural (they had the highest content of this compound). The aroma compounds, including 2-penten-1-ol, 2-propenyl 2-propenoic acid ester, (*E*,*E*)-2,4-heptadienal, verbenone (I), 2-propenoic acid, 3-phenyl-methyl ester, and 5-methyl-decane, were highly correlated with the plant-based note, whereas hexanoic acid was positively associated with acid odor. Moreover, a high correlation was observed between honey-like and sweet aroma with the phenylethyl acid, which has a characteristic floral note. Another correlation was observed between 2,6-dimethyl-nonane and yellow color. It can be concluded that the sugars were highly correlated with volatile compounds (Figure 3). Sucrose, fructose, and glucose were highly correlated with the 2,6-dimethyl-nonane, dimethyl disulfide, and nonanoic acid, respectively. Furthermore, a positive correlation was noted between sugar content and pungent, honey-like, and sweet odor. 

In addition, some correlations were observed between individual aroma compounds. A high correlation was demonstrated between 4-methyl-octane, 4-methyl-decane, and tetradecane, all being alkanes. The next group of positively correlated volatiles included 2,4-dimethyl-heptane, 2-methyl-nonane, and dodecane, which were also representatives of alkanes. It should be noticed that the sensory attributes can by no means be associated with an individual aroma compound. The unique sensory characteristics of the studied bee products were attributed to the superimposed and synergistic effects of volatile compounds [49], which in the case of bee products correspond mostly to the botanical and geographical origin [19,20].

## 3. Materials and Methods

### 3.1. Chemicals and Reagents

The mix of C6-C30 *n*-alkanes, d-(+)-glucose, d-(−)-fructose, sucrose, methanol, acetonitrile, and water was purchased from Sigma-Aldrich (St. Louis, MO, USA).

### 3.2. Research Material

Beeswax, beebread, bee pollen, and multiflorous honey were obtained from the same apiary from the Kujawy region (central of Poland) by a professional beekeeper. The samples were collected in 2019, packed in polypropylene bags, and kept refrigerated at 4 °C before analyzed.

### 3.3. Determination of Volatile Composition of Bee Products

The volatile analysis of bee products was carried out by the headspace solid phase microextraction gas chromatography mass spectrometry (HS-SPME GC/MS) according to the method developed by Plutowska et al. [50] with some modifications. Therefore, 2 g of each bee product were weighed into a 20-mL vial. The vials were placed on an Eppendorf agitator/heater (Germany), shaken and heated (40 °C, 50 min, 600 rpm), and then the volatiles were allowed to absorb onto the SPME fiber for 15 min at 50 °C (without shaking). A 50/30 µm stable DVB/CAR/PDMS fiber (Supelco, Bellefonte, PA, USA) was used. The injection was done manually. Thus, the SPME fiber was introduced into the chromatograph injection port (splitless mode), where the analytes were desorbed at 250 °C for 5 min and transferred onto a capillary column (DB-WAX, 30 m, 0.25 mm × 0.50 µm). The analyses were performed using a gas chromatograph (Agilent Technologies 7890A GC system, Santa Clara, CA, USA) coupled to a mass spectrometer (Agilent Technologies 5975C VL MSD system, Santa Clara, CA, USA). The temperature was initially set to 40 °C and held for 5 min. Then, it was increased to 200 °C and held for 1 min. Finally, it was increased to 240 °C and held for 5 min. In the method, helium was used as the carrier gas with a constant flow rate of 1 mL/min. 

The analyses were carried out in triplicate. The compounds were identified by comparing the obtained linear retention indices, retention times, and mass spectra with data from the Wiley Registry 7th Edition Mass Spectral Library (Wiley and Sons Inc., Weinheim, Germany) and the National Institute Standards and Technology (NIST) 2005 Mass Spectral Library. Linear retention indices (LRIs) were calculated using the C6-C30 *n*-alkanes mix (Sigma-Aldrich, MO, USA). The results were presented as the total peak area of the individual compounds identified in the database.

### 3.4. Determination of Sugar Content in Bee Products

About 0.5 g of bee product samples were dissolved in 5 mL of 70% methanol. Thus, the prepared solutions were boiled for 15 min. Afterwards, the samples were cooled to room temperature and next filtered through a PTFE syringe filter (pore size 0.45 μm). Additionally, before analysis by high performance liquid chromatography (HPLC), the sample was centrifuged (MPW-260R, Poland) for 10 min (7500 RPM) [51].

The contents of glucose, fructose, and sucrose were determined using a Sykam apparatus (Fürstenfeldbruck, Germany) equipped with a refractometric detector (RI) from the same manufacturer. The separation was carried out on a Cosmosil Sugar-D 250 × 4.6 column (Nacalai Tesque, INC; Kyoto, Japan) at 35 °C. The mobile phase consisted of acetonitrile and water (75:25, *v*/*v*), while, the elution was carried out in an isocratic gradient by 15 min with a flow rate of 1 mL/min. Sugars were identified based on the retention times of the available standards, and their contents were calculated based on the concentration of the respective standard and expressed as g/100 g sample [52].

### 3.5. Quantitative Descriptive Analysis (QDA) of Bee Products

The sensory quality of honey products, including beeswax, bee bread, pollen, and honey, was evaluated by the QDA method, according to ISO standard [53]. The assessment was carried out by a sensory panel (six persons–five women and one man) previously selected, trained, and monitored according to the ISO guidelines [54]. The panel determined the list of all descriptors of aroma and appearance for the evaluated samples. There were six attributes of odor (honey-like, sweet, acid, pungent, waxy, and plant-based) and one attribute of appearance–color, which was defined as a yellow color intensity according to a color pattern RAL 075 80 60—scale value 5 with light–dark scale edges. The attributes of odor had non intensive–very intensive scale edges.

The assessments were carried out at a sensory laboratory room, which fulfils the requirements of the ISO standards [55]. The sensory laboratory was a temperature and humidity-controlled room (21 °C, 40%), equipped with 10 individual boxes, computers, and appropriate lighting. A computerized sensory program FIZZ (Biosystemes, Counternon, France) was used for the evaluations, and for collecting results, followed by their statistical analysis and graphical presentation. 

### 3.6. Statistical Analysis

The data are presented as mean values ± standard deviations of triplicate measurements. The differences between samples were analyzed by a one-way ANOVA with Tukey’s multiple comparison test (*p* < 0.05) using STATISTICA 13.0 (StatSoft Inc., Tulsa, OK, USA).

The data from the sensory analysis was evaluated using FIZZ (Biosystemes, Counternon, France). Then, the ANOVA was conducted to determine which sensory attributes were statistically significant, and Tukey’s test was performed to show similarities and differences between the investigated products assessed by the sensory panel. 

PLS was fitted with the use of “plsdepot” package in R (version 4.0.4, R Foundation for Statistical Computing, Vienna, Austria).

## 4. Conclusions

As already mentioned, this is the first study characterizing the composition of volatile compounds in four different bee products (beeswax, beebread, bee pollen, and honey) collected in the same batch. The conducted study showed that each bee product possessed its own unique profile of volatiles. However, the volatiles present in bee pollen played an important role in creating the volatile compound profile of beebread. The bee pollen was characterized by the highest number of volatiles, when compared to the other bee products. Alkanes were the main compounds detected in the bee products (except for honey). Three volatile compounds (nonanal, benzaldehyde, and furfural) were present in all samples of beeswax, beebread, bee pollen, and honey. Moreover, the major volatiles in beeswax were 2,4-dimethyl-heptane, octanal, nonanal, and 4-methyl-octane; in beebread: Decane, 1-tridecane, and furfural; in bee pollen: 2,4-dimethyl-heptane, 4-methyl-octane, decane, acetic acid, and 5-methyl-decane, whereas in honey: Benzaldehyde, furfural, and linalool. Thereby, the content of sugars, color, and odor descriptors such as honey-like, sweet, and pungent, were positively correlated only in honey. Beeswax was related to a waxy odor, bee pollen to a plant-based aroma, and beebread to an acid aroma. Therefore, it can be concluded that the volatile profile as well as sugar content, and furthermore sensory analysis depend on the stage of their processing by bees. Moreover, the characteristic of the volatile and sensory profiles and sugar content in the bee products can help the broader use of these products in the food industry to develop new functional foods.

## Figures and Tables

**Table 1:** The profile of volatile compounds identified in bee products with their potential aroma descriptions and relative abundance presented.

No.	Compound	Aroma Description	LRI _exp._	LRI _lit._	Bee Products			
					Beeswax	Beebread	Bee Pollen	Honey
					Peak Areas [ppm]			
X1	2,4-dimethyl-heptane	-	789	797	26.07 ± 2.47 ^a B^	12.29 ± 0.06 ^d C^	36.73 ± 2.94 ^a A^	-
X2	4-methyl-octane	-	822	823	12.56 ± 0.19 ^fg BC^	6.42 ± 0.64 ^efg C^	36.98 ± 4.42 ^a A^	-
X3	nonane	Alkane *	899	900	7.60 ± 0.45 ^e A^	4.50 ± 0.38 ^ghjk B^	-	-
X4	3,8-dimethyl-decane	-	948	MS	-	5.35 ± 0.47 ^fghi A^	5.02 ± 0.04 ^f A^	-
X5	2-methyl-nonane	-	954	960	9.75 ± 0.17 ^de A^	8.07 ± 0.57 ^ef B^	6.59 ± 0.47 ^f B^	-
X6	decane	-	1000	1000	-	22.55 ± 0.34^a A^	23.49 ± 3.40 ^bc A^	-
X7	4-methyl-decane	-	1001	1005	3.99 ± 0.20 ^fg A^	4.57 ± 0.35 ^ghij A^	4.11 ± 0.59 ^f A^	-
X8	2,6-dimethyl-nonane	-	1010	1014	-	15.05 ± 0.82 ^bc A^	6.50 ± 0.08 ^f B^	-
X9	2,6,11-trimethyl-dodecane	-	1012	1015	-	23.92 ± 0.63 ^a A^	17.95 ± 3.10 ^cd A^	-
X10	α-pinene	pine, turpentine *	1029	1030	3.14 ± 0.32 ^fgh^	-	-	-
X11	5-methyl-decane	-	1059	MS	-	2.95 ± 0.08 ^hijkl B^	19.61 ± 0.47 ^bc A^	-
X12	dimethyl disulfide	sulfurous *	1087	1094	-	2.92 ± 0.44 ^hijkl A^	-	2.08 ± 0.24 ^h A^
X13	hexanal	green, fruity **	1090	1093	-	13.71 ± 1.46 ^cd A^	4.03 ± 0.00 ^f B^	
X14	benzyl methyl sulfoxide	-	1102	MS	-	-	24.94 ± 3.28 ^b^	-
X15	1-(3,3-dimethylbutyl)benzene	-	1120	MS	-	1.83 ± 0.07 ^jkl A^	2.48 ± 0.35 ^f A^	-
X16	2,6,10-trimethyl-tetradecane	-	1190	MS	-	2.57 ± 0.05 ^jkl A^	2.77 ± 0.07 ^f B^	-
X17	dodecane	alkane *	1225	1227	10.14 ± 0.44 ^de B^	2.79 ± 0.20 ^ijkl C^	12.79 ± 0.16 ^de A^	-
X18	nonadecane	alkane *	1265	1263	2.81 ± 0.24 ^fgh B^	5.98 ± 0.66 ^efg A^	-	-
X19	styrene	pungent **	1270	1273	-	2.75 ± 0.05 ^ijkl A^	2.72 ± 0.10 ^f A^	-
X20	*o*-cymene	-	1275	1276	-	-	-	2.39 ± 0.29 ^gh^
X21	octanal	aldehydic *	1291	1291	18.60 ± 0.41 ^b A^	1.90 ± 0.21 ^jkl B^	0.70 ± 0.01 ^f C^	-
X22	*p*-cymene	sweet	1293	1292	-	-	-	13.67 ± 2.10 ^d^
X23	2-penten-1-ol	fruity	1315	1314	-	-	1.42 ± 0.01 ^f^	-
X24	6-methyl-5-hepten-2-one	green, citrus	1342	1341	-	4.40 ± 0.18 ^ghijk A^	2.38 ± 0.08 ^f B^	-
X25	1-tridecene	pleasant	1344	1342	-	22.09 ± 0.45 ^a A^	0.72 ± 0.02 ^f B^	-
X26	2-propenyl 2-propenoic acid, ester	-	1456	MS	-	-	1.52 ± 0.02^f^	-
X27	tetradecane	alkane *	1399	1400	1.91 ± 0.14 ^fgh A^	2.51 ± 0.37 ^jkl A^	1.76 ± 0.04 ^f A^	-
X28	nonanal	aldehydic *	1416	1418	15.18 ± 1.75 ^c A^	1.63 ± 0.18 ^l B^	2.78 ± 0.04 ^f B^	3.44 ± 0.12 ^gh B^
X29	1-heptanol	green *	1458	1459	1.55 ± 0.01 ^fgh^	-	-	-
X30	*cis*-linalool oxide	flower, woody	1462	1461	-	-	-	2.43 ± 0.10 ^gh^
X31	acetic acid	sour, pungent	1468	1470	-	17.37 ± 1.34 ^b A^	20.08 ± 3.41 ^bc A^	3.57 ± 0.02 ^gh B^
X32	furfural	bready *	1477	1476	2.08 ± 0.15 ^fgh C^	24.07 ± 2.06^a B^	3.87 ± 0.04 ^f C^	37.19 ± 1.51 ^b A^
X33	2-ethyl-1-hexanol	citrus *	1490	1493	1.54 ± 0.02 ^fgh B^	-	-	2.02 ± 0.01 ^h A^
X34	(*E*,*E*)-2,4-heptadienal	-	1497	MS	-	-	0.81 ± 0.06 ^f^	-
X35	decanal	aldehydic *	1499	1500	13.18 ± 1.01 ^c^	-	-	-
X36	(*E*,*E*)-3,5-octadien-2-one	-	1545	MS	-	1.92 ± 0.11 ^jkl B^	2.19 ± 0.04 ^f A^	-
X37	benzaldehyde	almond-like	1558	1562	4.32 ± 0.13 ^f B^	5.55 ± 0.96 ^fgh B^	6.05 ± 0.88 ^f B^	52.39 ± 1.74 ^a A^
X38	1-octanol	waxy *	1561	1561	1.35 ± 0.04 ^fgh^	-	-	-
X39	linalool	floral, sweet	1566	1565	-	-	-	28.79 ± 1.09 ^c^
X40	1-ethyl-1*H*-pyrrole-2-carbaldehyde	burnt, roasted	1612	1616	-	1.58 ± 0.01 ^l A^	0.93 ± 0.07 ^f B^	-
X41	hotrienol	sweet tropical *	1633	1632	-	-	-	8.45 ± 0.14 ^ef^
X42	dihydro-4-methyl-2(3*H*)-furanone	-	1649	1653	-	-	-	9.17 ± 0.09 ^e^
X43	1- nonanol	floral *	1660	1668	0.85 ± 0.01 ^h^	-	-	-
X44	butyrolactone	sweet, caramel **	1677	1673	-	1.81 ± 0.39 ^kl A^	2.24 ± 0.26 ^f A^	-
X45	2/3-methyl-butanoic acid	sweaty	1682	1684	-	1.20 ± 0.14 ^l B^	-	14.91 ± 1.15 ^d A^
X46	2-methyl-hexanoic acid	fruity	1755	1757	-	11.43 ± 1.05 ^d A^	4.67 ± 0.23 ^f B^	-
X47	verbenone (I)	minty, spicy **	1732	1733	-	-	0.66 ± 0.03 ^f^	-
X48	3-methylvaleric acid	animal *	1823	1826	-	-	-	2.91 ± 0.01 ^gh^
X49	hexanoic acid	fatty *	1844	1842	-	8.51 ± 0.13 ^e A^	6.70 ± 0.02 ^ef B^	5.27 ± 0.02 ^fg C^
X50	*p*-cymen-8-ol	floral, sweet **	1879	1872	-	-	-	2.59 ± 0.06 ^gh^
X51	benzyl alcohol	sweet, floral *	1900	1906	0.98 ± 0.01 ^gh B^	-	-	2.09 ± 0.01 ^gh A^
X52	phenylethyl alcohol	floral *	1923	1931	0.69 ± 0.01 ^h B^	-	-	2.47 ± 0.01 ^gh A^
X53	heptanoic acid	cheesy *	1991	1990	-	-	-	2.84 ± 0.01 ^gh^
X54	2-propenoic acid, 3-phenyl-, methyl ester	fruity	2056	2065	-	-	0.92 ± 0.11 ^f^	-
X55	nonanoic acid	waxy *	2200	2202	-	1.21 ± 0.25 ^l B^	-	3.02 ± 0.02 ^gh A^
sum					138.29 ± 5.73 ^C^	245.40 ± 1.70 ^A^	267.13 ± 8.32 ^A^	201.70 ± 3.39 ^B^

* www.flavornet.org (accessed on 12 May 2021); ** pherobase; LRI: Linear retention indices (exp.: Experimental, lit.: Literature); MS: Compounds identified according to their mass spectrum. Values were presented as mean ± standard deviation. ^a–l^ Values followed by different letters in the same column are significantly different (*p* < 0.05) and ^A–C^ values followed by different letters in the same row are significantly different (*p* < 0.05) as determined by Tukey’s multiple comparison test.

**Table 2:** The content of sugars (fructose, glucose, and sucrose at the level of g/100 g), total amount of sugars, and fructose/glucose ratio (F/G) determined in bee products.

Bee Products	Sugar [g/100 g]				
	Fructose	Glucose	Sucrose	TOTAL Amount of Sugars	F/G Ratio
Beeswax	0.06 ± 0.00 ^d^	2.03 ± 0.14 ^d^	-	2.09	0.03
Beebread	11.58 ± 0.12 ^c^	5.12 ± 0.33 ^c^	0.11 ± 0.00 ^a^	16.81	2.26
Bee pollen	14.06 ± 0.01 ^b^	11.56 ± 0.43 ^b^	0.01 ± 0.00 ^b^	25.63	1.22
Honey	27.60 ± 0.48 ^a^	26.32 ± 0.49 ^a^	0.10 ± 0.01 ^a^	54.02	1.05

Values were presented as mean ± standard deviation. Values followed by different letters in the same column are significantly different (*p* < 0.05), as determined by Tukey’s multiple comparison test.

## Data Availability

Not applicable.
